# Metagenomic Methods for Addressing NASA's Planetary Protection Policy Requirements on Future Missions: A Workshop Report

**DOI:** 10.1089/ast.2022.0044

**Published:** 2023-08-14

**Authors:** Stefan J. Green, Tamas Torok, Jonathan E. Allen, Emiley Eloe-Fadrosh, Scott A. Jackson, Sunny C. Jiang, Stuart S. Levine, Shawn Levy, Lynn M. Schriml, W. Kelley Thomas, Jason M. Wood, Scott W. Tighe

**Affiliations:** ^1^ Genomics and Microbiome Core Facility, Rush University Medical Center, Chicago, Illinois, USA.; ^2^ Ecology Department, Lawrence Berkeley National Laboratory, Berkeley, California, USA.; ^3^ Lawrence Livermore National Laboratory, Livermore, California, USA.; ^4^ DOE Joint Genome Institute, Lawrence Berkeley National Laboratory, Berkeley, California, USA.; ^5^ National Institute of Standards and Technology, Gaithersburg, Maryland, USA.; ^6^ Department of Civil and Environmental Engineering, University of California, Irvine, California, USA.; ^7^ MIT BioMicro Center, Massachusetts Institute of Technology, Cambridge, Massachusetts, USA.; ^8^ HudsonAlpha Institute for Biotechnology, Huntsville, Alabama, USA.; ^9^ Institute for Genome Sciences, University of Maryland School of Medicine, Baltimore, Maryland, USA.; ^10^ Hubbard Center for Genome Studies, University of New Hampshire, Durham, New Hampshire, USA.; ^11^ Research Informatics Core, University of Illinois at Chicago, Chicago, Illinois, USA.; ^12^ Vermont Integrative Genomics, University of Vermont, Burlington, Vermont, USA.

**Keywords:** Metagenomics, Planetary protection, Contamination, Spacecraft Assembly Facility, DNA

## Abstract

Molecular biology methods and technologies have advanced substantially over the past decade. These new molecular methods should be incorporated among the standard tools of planetary protection (PP) and could be validated for incorporation by 2026. To address the feasibility of applying modern molecular techniques to such an application, NASA conducted a technology workshop with private industry partners, academics, and government agency stakeholders, along with NASA staff and contractors. The technical discussions and presentations of the Multi-Mission Metagenomics Technology Development Workshop focused on modernizing and supplementing the current PP assays. The goals of the workshop were to assess the state of metagenomics and other advanced molecular techniques in the context of providing a validated framework to supplement the bacterial endospore-based NASA Standard Assay and to identify knowledge and technology gaps. In particular, workshop participants were tasked with discussing metagenomics as a stand-alone technology to provide rapid and comprehensive analysis of total nucleic acids and viable microorganisms on spacecraft surfaces, thereby allowing for the development of tailored and cost-effective microbial reduction plans for each hardware item on a spacecraft. Workshop participants recommended metagenomics approaches as the only data source that can adequately feed into quantitative microbial risk assessment models for evaluating the risk of forward (exploring extraterrestrial planet) and back (Earth harmful biological) contamination. Participants were unanimous that a metagenomics workflow, in tandem with rapid targeted quantitative (digital) PCR, represents a revolutionary advance over existing methods for the assessment of microbial bioburden on spacecraft surfaces. The workshop highlighted low biomass sampling, reagent contamination, and inconsistent bioinformatics data analysis as key areas for technology development. Finally, it was concluded that implementing metagenomics as an additional workflow for addressing concerns of NASA's robotic mission will represent a dramatic improvement in technology advancement for PP and will benefit future missions where mission success is affected by backward and forward contamination.

## 1. Introduction

Since the beginning of extraterrestrial exploration by NASA, planetary protection (PP) has been an important effort to prevent biological forward contamination of non-Earth environments. The Committee on Space Research (COSPAR) has formulated a Planetary Protection Policy with associated implementation requirements as an international standard to protect against interplanetary biological and organic contamination (UNOOSA, 2017).

Bacterial endospores, due to their ability to survive extreme conditions, are considered worst-case biological indicators for verifying spacecraft cleanliness (COSPAR, 2011). The current NASA Standard Assay (NSA) is designed to enumerate microorganisms that have formed endospores at the time of sampling, respire aerobically, survive a heat treatment at 80°C for 15 min, and can be cultivated on Tryptic Soy Agar (NASA, 2011).

Although the NSA method, developed to assess PP risk for spacecraft bound for Mars, has been suitable for some flight missions (*e.g.*, COSPAR Category IVa, IVb and IVc to Mars), the NSA method alone is insufficient for missions with sample return or life detection. The inability of the NSA method to detect all PP-relevant taxa or dead organisms or environmental DNA (eDNA) could potentially lead to false negative results for undetected biology. Although the NSA may serve as an indicator of biological cleanliness, it does not identify PP-relevant microbial taxa capable of flight survival, nor can it address the issue of microbial diversity on spacecraft surfaces.

Bacterial endospore counts are neither representative of total spacecraft bioburden nor the only microorganisms of PP concern (*e.g.*, La Duc *et al.*, 2007; Cooper *et al.*, 2011; Hendrickson *et al.*, 2021) and not all recovered organisms, viruses, or virus like particles (VLP), recovered from extreme environments require sporulation to survive. To assess total bioburden and to identify microorganisms of concern, as well as to understand their true potential for forward contamination during robotic spaceflight missions, cultivation-independent molecular methods capable of detecting all microorganisms are needed (NASA, 2019).

Although PP requirements have evolved to meet the needs of increasingly sophisticated missions, nucleic acids-based technologies have not yet been vetted and adopted into standard PP practices except as research tools (La Duc *et al.*, 2014). Molecular biology approaches targeting nucleic acids enable broad-based detection of all microbial taxa and deliver information needed for life detection protocols. Such information can be used to develop tailored microbial reduction strategies, to communicate biological contamination and function risk assessments, and to facilitate options for end-of-mission strategies.

For example, in Mars sample return missions, detailed inventories of spacecraft bioburden are essential when comparing outbound and inbound samples. Such analyses are needed to fully and confidently assess the authenticity of returned and *in situ* analyzed samples (*i.e.*, bio-signatures of extraterrestrial origin vs. terrestrial contamination) (Venkateswaran *et al.*, 2012; Minich *et al.*, 2018; Hendrickson *et al.*, 2021). For Ocean Worlds missions, there is an increase in the cleanliness standard for PP compliance, and more complete spacecraft bioburden data will be needed for probabilistic risk assessments for landing and sampling.

Addressing the Space Studies Board's (SSB) earlier recommendations, research performed in FY19 demonstrated that sample processing has advanced sufficiently, and NASA's future missions can now implement molecular biology methods. Appropriate and stringent validation will still be necessary for effective implementation (Venkateswaran *et al.*, 2012). The high informational content of metagenomic sequence data for species- and strain-level taxonomic assignment and for functional gene identification allows for an informed risk-based assessment of spacecraft biological contamination.

In addition, taxon- or gene-specific bioburden can be quantitatively determined using quantitative PCR techniques (*e.g.*, Schnetzinger *et al.*, 2013). Implementation of metagenomics from the current research-focused applications to routine PP practice will benefit all current and planned future missions. It will increase confidence in the ability to reduce forward contamination threats during exploration of planets, and it will lead to tailored, more efficient, and cost-effective microbial reduction plans for each hardware item on a spacecraft. Worst-case scenario modeling approaches (Singh *et al.*, 2018) will be avoided by characterizing the total microbial community on each piece of hardware.

Although remarkable strides have been made in molecular biology applications, there are still components of the overall workflow that require tailoring for spacecraft monitoring. These include: (1) sample collection; (2) sample processing (extraction, library preparation, and sequencing); (3) sequence data processing and bioinformatics; and (4) establishing a curated list of microorganisms and genes relevant to each mission.

To address the evolving technologies and to support the needs of the PP program, the workshop was held to facilitate a state-of-the-art assessment of metagenomics and to provide NASA with recommendations for a modern nucleic acids-based spacecraft cleanliness verification approach.

## 2. Objectives

The primary objective of this article is to summarize the outcomes of the Multi-Mission Metagenomics Technology Development Workshop held on January 19, 2022. The workshop goals were to (1) assess the state of metagenomics and other advanced molecular biology techniques in the context of providing a validated framework to supplement the NSA, (2) identify knowledge and technology gaps, and (3) determine what steps are needed to certify molecular techniques as new PP methods.

Although shotgun metagenomic sequencing and other cultivation-independent techniques have been widely adopted in academia, industry, and government agencies, there is a requirement to validate these methods to address the SSB recommendations and to modernize NASA's PP practices (National Academies of Sciences, Engineering, and Medicine, 2021). The steps in certification include sample collection and processing, nucleic acids-based molecular analyses, data analysis pipeline development, and quantitative risk assessment for PP-relevant microorganisms.

The workshop highlighted low biomass sampling, reagent contamination, and inconsistent bioinformatic data analysis as critical areas for further knowledge base and technology development.

The PP implementation and spacecraft cleaning workflows and the resulting high cleanliness make molecular characterization of low biomass spacecraft surfaces a challenge. Nonetheless, experts at this workshop suggested that nucleic acids-based technologies have matured, and steps should be taken to initiate and adopt such techniques for spacecraft bioburden taxonomic assessment, functional gene/functional potential assessment, and quantitative analyses of taxa and genes of interest.

The potential benefits of achieving the workshop goals for current and planned future NASA missions include: (1) the ability to perform rapid assessment of multi-domain (*i.e.*, Bacteria, Archaea, and Eukarya) spacecraft-associated microbial burden; (2) increased throughput by automation; (3) data that will contribute to risk analysis and modeling based on functional profiles rather than conservative guidelines; and (4) the design of highly tailored microbial reduction strategies used to reduce relevant viable microorganisms present on hardware.

## 3. Current Technologies for Bioburden Assessment

Historically, bioburden assessment and microbial reduction strategies were adopted in part from food and pharmaceutical practices. The current NSA technique was established in the 1960s (NASA, 1967), though subsequently it was reported that the microorganisms cultured by the NSA account for <0.1% of the overall microbial population present on a given spacecraft surface (Ghosh *et al.*, 2010).

Further, not all endospore-forming bacteria tolerate spaceflight conditions, whereas other microorganisms of PP relevance, such as fungi, will be missed due to the restrictive cultivation conditions of the NSA (*e.g.*, La Duc *et al.*, 2007; Cooper *et al.*, 2011). As spacecraft size and complexity increases, including surfaces that are difficult to clean, along with the use of probabilistic risk assessment-based requirements (*e.g.*, Mars 2020 sample return and Europa Lander), the assumption that potentially all microorganisms may tolerate spaceflight is untenable from biological, engineering, and mathematical perspectives.

Thus, taxonomic and functional gene content information derived from molecular methods (*e.g.*, metagenomic sequencing) is the only way to populate the kinds of risk models available for identification and quantitation of PP-relevant (*i.e.*, contamination threat) microorganisms.

In addition to the NSA, NASA has certified other bioburden assessments, including two rapid assays approved in 2005 as alternative measures of microbial contamination (Table 1). The adenosine triphosphate (ATP) assay and the Limulus Amoebocyte Lysate (LAL) assay (Benardini and Venkateswaran, 2016) were implemented principally to not only provide rapid analysis to circumvent the 3-day incubation period of the NSA, but also offer a means to screen spacecraft hardware for microbial contamination (Kern *et al.*, 2005; Morris *et al.*, 2010).

**Table 1.** Comparison of NASA-Approved Methods for Bioburden Assessment of Spacecraft Surfaces

Methods	NASA implemented	Analysis targets	Microbial diversity*^a^*	Quantification	Turnaround time	Cost	Background contamination risk
NSA	Since 1970s	Culturable bacterial endospores	No	Absolute	72–96 h	$^b^	Low
ATP assay	Since 2010^c^	Metabolizing cells	No	Absolute	∼30 min	$$	Medium
LAL assay	Since 2010^c^	Endotoxin producing cells	No	Absolute	∼30 min	$$	Medium

^a^ Microbial diversity assessment capability.

^b^ Labor cost is not included and when labor is considered, NSA is more expensive than ATP and LAL assays.

^c^ Since 2010 during and after Mars Science Laboratory.

ATP = adenosine triphosphate; LAL = Limulus Amoebocyte Lysate; NSA = NASA Standard Assay.

However, the LAL and ATP assays are unable to provide any taxonomic information, to distinguish between microorganisms of PP concern and those of no concern, or to provide quantitative cell abundance for microorganisms of concern (Table 1). Thus, the lack of a rapid quantitative or semi-quantitative method for assessing microbial diversity and taxonomy is recognized as a capability gap that still needs to be addressed to characterize total and PP-relevant microorganisms in cleanrooms and on spacecraft. Analyses of nucleic acids, including metagenomic sequencing and quantitative digital PCR analysis, are the most appropriate technologies to address this gap.

## 4. Developing and Implementing Molecular Biology Techniques for Microbial Inventory of Low Biomass Environments

In 1992, the SSB document “Biological Contamination of Mars: Issues and Recommendations” (NRC, 1992) asked for the immediate development of methods beyond the NSA for determining microbial bioburden. In 2006, in a document titled “Preventing the Forward Contamination of Mars” (NRC, 2006), the SSB recommended that NASA require the systematic collection of phylogenetic data to assess microbial communities in the assembly, test, and launch operation (ATLO) environments associated with spacecraft to be sent to Mars.

This 2006 report also recommended the implementation of broad-based assessments of microbial bioburden such as the ATP and LAL assays. Further, in 2019, the NASA Planetary Protection Independent Review Board (PPIRB) prepared a final report to NASA/Science Mission Directorate (SMD) that indicated that NASA PP policy should move beyond exclusive adherence to bacterial endospore counts. The use of proven modern techniques and well-established genomic tools for monitoring and characterization of bioburden of cleanroom facilities and flight hardware was encouraged (Space Science Board, 2018, 2020).

The Mars Program Office at JPL and NASA's Office of Planetary Protection funded several activities to test molecular methods and the feasibility of such methods for measuring bioburden and biodiversity of spacecraft associated surfaces. Despite these recommendations, however, the NSA remains the only fully validated and approved method of spacecraft bioburden assessment for verifying compliance with COSPAR.

Over the past two decades, the rate of molecular tool development for non-cultivation-based characterization of microbial communities has increased dramatically. These techniques have included low-throughput 16S ribosomal RNA (rRNA) gene amplicon cloning (late 1990s), 16S rRNA microarray analyses (early 2000s; *e.g.*, Loy *et al.*, 2002), high-throughput 16S rRNA gene amplicon and fungal internal transcribed spacer region amplicon next-generation sequencing (*e.g.*, Huse *et al.*, 2007; Buee *et al.*, 2009), and shotgun metagenomic sequencing in late 2000s and 2010s (*e.g.*, Bashir *et al.*, 2016; Danko *et al.*, 2021).

High-throughput 16S rRNA gene amplicon sequencing has been employed for a large number of studies of microbial communities but the utility of the method is negatively impacted by incomplete taxonomic coverage/lower diversity (*e.g.*, Klindworth *et al.*, 2013; Hug *et al.*, 2016), limited phylogenetic resolution (*e.g.*, Poretsky *et al.*, 2014), and biases introduced by PCR amplification leading to a distortion of the true microbial community structure (*e.g.*, Green *et al.*, 2015; Naqib *et al.*, 2019).

Further, no single primer set is capable of targeting all domains of life. Conversely, non-targeted shotgun metagenomic sequencing provides the best opportunity for low-bias preparation of low biomass samples. The non-selective nature of metagenomics allows it to serve as a discovery tool with potential for detecting and identifying PP-relevant microbial taxa across all three domains of life and from some viruses, regardless of prior identification or ability to cultivate.

Although some metagenome sequencing approaches are relatively rapid, the overall turn-around time for nucleic acids extraction, library preparation, sequencing, and analysis is not rapid enough for routine spacecraft bioburden assessment. A comparison of relevant characteristics of real-time PCR, digital PCR, amplicon sequencing, and shotgun sequencing is shown in Table 2.

**Table 2.** Comparison of Proposed Molecular Methods for Bioburden Assessment of Spacecraft Surfaces

Methods	Analysis targets	Microbial diversity	Quantification	Previous NASA work	Turnaround time for data and analysis	Cost	Background contamination risk
Molecular methods for bioburden measurements							
Real-time quantitative PCR	Variable, depending on primers	No^a^	Absolute or relative	Yes	<24 h	$	Variable, depending on assay specificity
Digital quantitative PCR	Variable, depending on primers	No^a^	Absolute	No	<24 h	$$	Variable, depending on assay specificity
Molecular methods for microbial diversity assessments							
NGS Amplicon sequencing	Variable, depending on primers	Yes	Relative, with possibility of absolute	Yes	∼1 week	$$	High
NGS metagenome sequencing	All dsDNAs^b^	Yes	Relative, with possibility of absolute	Yes	∼1 week	$$$	High

^a^ Assay specificity can provide evidence of specific taxa.

^b^ Most metagenome library synthesis is performed using protocols targeting double-stranded DNA; however, assays targeting single- and double-stranded DNA are available.

dsDNA; NGS.

To overcome the turnaround time limitations of sequencing methods, the participating workshop scientists identified quantitative PCR as a viable option, including both real-time PCR and digital PCR. Digital PCR has several advantages relative to real-time PCR, including absolute quantification without need for standards, relative insensitivity to enzymatic inhibitors, and robust resolution at low template copy numbers (*e.g.*, Tan *et al.*, 2021).

For samples with low levels of biomass, severe patchiness can occur, and numerous replicate extractions and analyses can be employed to detect contamination. Although digital PCR has been suggested for low yield samples, its use has not yet been widely adopted by NASA researchers.

Singly or in combination, the use of these molecular approaches could enable whole genome data acquisition and phylogenetically informed quantitative results more rapidly than the three days of cultivation needed for NSA. Digital PCR performed with targeted molecular assays could provide quantitative data relevant to PP cleanliness concerns within a single day. Assays targeting highly conserved genes (*e.g.*, 16S rRNA genes in bacteria and archaea) are likely to be more sensitive (*e.g.*, due to the presence of multiple copies of rRNA gene operons in most microbial cells).

However, varying rRNA gene copy numbers cannot be converted exactly into cell numbers, though lineage-specific conversions have been attempted (*e.g.*, Kembel *et al.*, 2012; Angly *et al.*, 2014). For narrower taxon-specific analyses, single copy genes (*e.g.*, *rpoB*, *gyrB*, etc.) are easier to convert to cell numbers and may also be relatively insensitive to reagent contamination issues that afflict non-targeted metagenomics approaches. Quantitative analyses should be employed in tandem with metagenomic sequencing to provide high-resolution functional gene content and allow for discovery of novel microorganisms of PP concern.

These combined datasets will provide quantitative and semi-quantitative taxonomic and functional information about spacecraft-associated microbiomes, enabling decision making on a comprehensive data set and thereby lowering contamination risk.

Although the focus of committee members was on PP rather than Astrobiology, the committee did discuss the need to differentiate between live cells (affecting forward contamination) and dead cells and viruses, VLP and eDNA molecules (affecting life detection science missions). Nucleic acids-based characterization of microbial communities can permit the differentiation of live and dead organisms (*e.g.*, Vaishampayan *et al.*, 2013), and this can be critically important, depending on mission criteria.

Although microbial growth and activity has been inferred from metagenomic sequence data using bioinformatic strategies (Emiola and Oh, 2018; Danko *et al.*, 2021), more commonly, a selective degradation or inactivation of free nucleic acid is performed through enzymatic (*e.g.*, DNase; Marotz *et al.*, 2018) or chemical (*e.g.*, propidium monoazide [PMA]; Nocker *et al.*, 2006) treatment. However, whether these methods are viable at the low nucleic acid levels in cleanroom and spacecraft samples is not yet known.

Further, prior studies have indicated that the application of PMA treatment may not generate quantitative results in complex ecosystems (Wang *et al.*, 2021). Alternatively, interrogation of RNA instead of DNA focuses sequencing efforts on viable organisms, as RNA molecules degrade rapidly after cell death (*e.g.*, Li *et al.*, 2017). Targeting RNA instead of DNA could also increase the limit of detection due to higher numbers of ribosomes per cell relative to copies of rRNA gene operons per cell (*e.g.*, Nardello *et al.*, 2020), though ribosome copy numbers cannot be readily converted into cell numbers.

## 5. Process Requirements for Validation of Metagenomic Sequence Technology

Although there are clear advantages to using molecular tools for evaluating spacecraft bioburden, these tools have specific limitations that must be addressed before a robust protocol can be implemented by NASA. The most serious of these limitations is derived from the extremely low nucleic acid yields from spacecraft swab samples.

These DNA recoveries are often in the femtogram (fg) to picogram (pg) range, which are levels at or below the tolerance threshold for established sequencing detection (*e.g.*, Rinke *et al.*, 2016). Further, the presence of reagent nucleic acid contamination must be considered when analyzing ultra-low bioburden samples. Ascertainment of the true signal from the background noise, as well as establishing reproducibility from multiple extractions (*e.g.*, de Goffau *et al.*, 2018), will be necessary.

Such low concentrations of recovered nucleic acids may require additional DNA amplification strategies (Silander and Saarela, 2008; Ahsanuddin *et al.*, 2017), which may lead to further complications from the amplification of the contaminating DNA within reagents (*i.e.*, the “kitome”) and other background signals as well as the potential of added noise from erroneous amplifications.

Reagent contamination issues can only be controlled through use of proper controls and ultrapure reagents (*e.g.*, Salter *et al.*, 2014; Eisenhofer *et al.*, 2019). In addition, although library synthesis in protocols can be largely bias-free, the nucleic acid extraction process is known to introduce bias, and microorganisms undergoing incomplete lysis can be undercounted or missed entirely in molecular analyses. Further, by itself, sequencing performed on extracted DNA cannot distinguish between DNA derived from living cells and remnant or contaminating DNA.

Thus, careful consideration is needed in the application of metagenomics to ensure that the appropriate protocols are developed to capture microbial signatures exclusively from living cells, as well as proper controls to account for background reagent-contaminating DNA “noise.” Additional challenges for deriving absolute quantification from sequencing may be overcome by implementing spike-ins during sample processing or by implementing quantitative rapid molecular methods such as digital quantitative PCR.

Nonetheless, in addition to extraction methods with complete microbial cell lysis, all methods will need ultra-clean reagents (*i.e.*, DNA free) across all stages of sample processing (*i.e.*, collection, extraction, pre-amplification, and library synthesis), proper controls, and stable bioinformatics pipelines.

Thus, a molecular verification process to supplement or replace the NSA bioburden assessment will have four key features: (1) rapid quantification of biological contaminants above background noise; (2) characterization of taxonomic and functional gene content of microorganisms; (3) viability assessment of detected microorganisms; and (4) accurate determination of the functional potential and risk profile of contaminants.

The first two features can be addressed with a molecular biology workflow, the third with chemical pretreatment followed by molecular analysis or direct interrogation of RNA in place of DNA, and the fourth can be addressed with bioinformatics. To achieve these goals, method development and validation is needed in four areas, including: (1) sample collection, (2) sample processing and sequencing, (3) bioinformatics, and (4) risk assessment.

### 5.1. Sample collection

**Recommendation:** Flight-certified swab and wipe methods for molecular assays are needed. Sampling device qualification should include a comparison of accuracy, precision, specificity, limit of detection, robustness, linearity, and background nucleic acid levels. A standard operating protocol for sample collection should be developed.

The certification goal is to secure flight-approved sampling devices for large (≥0.1 m^2^, wipe) and small (∼25 cm^2^, swab) surface areas that will have (1) repeatable and high yield microbial recovery from spacecraft surfaces; (2) optimized yield for release of biological materials from the sampling device; (3) molecular cleanliness with low noise/background nucleic acids and contact residue (<0.02 μg/cm^2^); (4) a predictable dynamic range of collection in high and low biomass systems (*i.e.*, those producing ≤10 ng of DNA and those producing >10 ng of DNA); (5) compliance with ISO5 to 8.5 cleanroom particulate count [foreign object debris (AIA/NAS 412)/ISO 14664-1:2015(E)]; and (6) compliance with electrostatic discharge demands (<50 V sensitivity; ANSI/ESDA/JEDEC JS-002-2014).

Prior studies have shown cotton swabs to be superior to Copan swabs for smaller (25 cm^2^) spacecraft surfaces for collecting endospore-forming bacteria (*e.g.*, Kwan *et al.*, 2011). Likewise, DNA-free macrofoam-based device collected more biomolecules when compared with the polyester wipes for larger (1 m^2^) surfaces. These studies employed quantitative PCR to measure microbial burden (Kwan *et al.*, 2011; Bargoma *et al.*, 2013).

If the goals of the metagenomics workshop are achieved, standardized sampling devices and an operating protocol for the collection of microorganisms and other bio-signatures from spacecraft surfaces will be developed along with a comprehensive background nucleic acids profile for the sampling devices. Efficiency of nucleic acids recovery and limits of detection will need to be evaluated with reference materials such as mock microbial communities. Procedures for approval testing and validation of new sampling devices should also be established.

### 5.2. Sample processing

Prior evaluation of sample processing techniques revealed that filtration devices increased the yield of the biomolecules of interest when low-biomass samples such as spacecraft-associated surfaces were examined (La Duc *et al.*, 2009, 2012). In addition, analysis of microbial communities using targeted amplicon sequencing suggested that microbial profiles were dominated by the kitome (Minich *et al.*, 2018). To gain information on the functional characteristics of spacecraft and cleanroom microbiomes, shotgun metagenome sequencing was also studied, including on samples with extremely low nucleic acid yields (*e.g.*, Danko *et al.*, 2021).

**Recommendation:** The sample processing protocol should meet the following requirements: (1) lyse open cells and permit nucleic acid isolation from all domains of life including hardy microorganisms; (2) differentiate between viable and non-viable taxa; (3) have the ability to process nucleic acid samples from both high and low biomass samples (*e.g.*, 1–10 and >10 pg); and (4) define robust sampling and library preparation negative and positive controls.

**FIG. 1.**
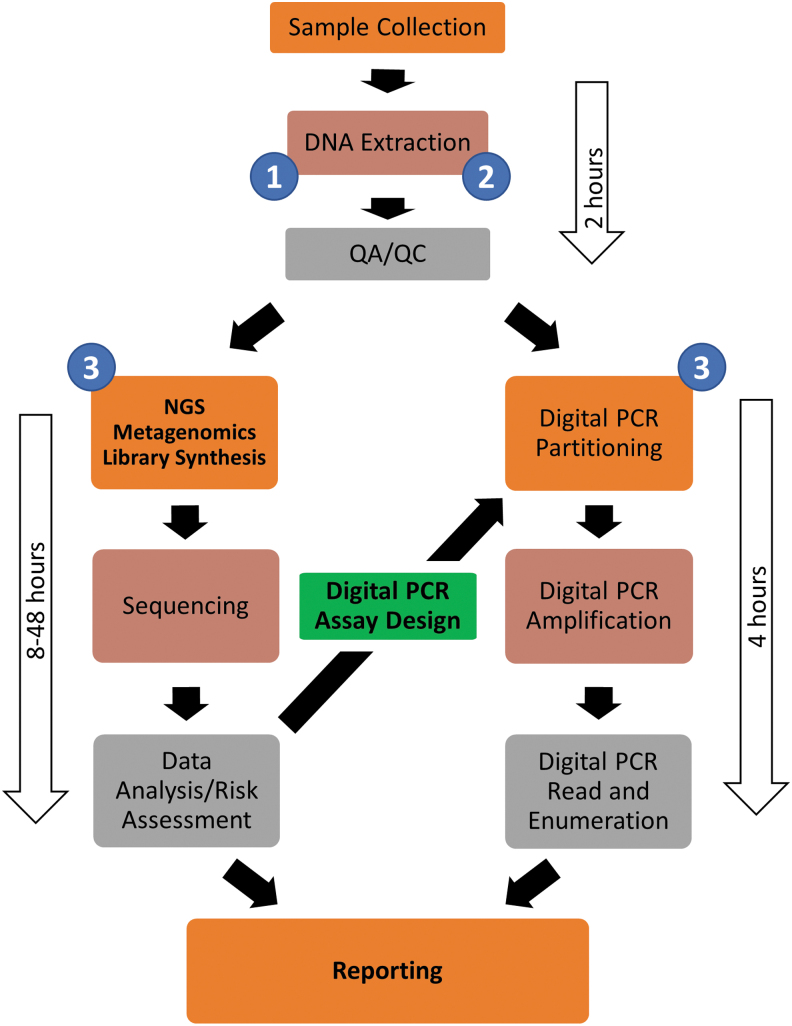
A proposed hybrid molecular biology workflow for PP bioburden assessment. A combination of rapid, multiplexed, sequence-guided quantitative digital PCR assays is proposed together with longer-term knowledge-building next-generation sequence-acquisition workflows. Digital PCR analysis, based on assays built on *a priori* knowledge of taxa and genes of interest, can be used for functional gene or rRNA gene targets. To address reproducibility issues and DNA extraction efficiency limitations, mixtures of microbial cells at known abundances can be spiked into DNA extractions (1). For viability testing, samples can be treated using PMA before DNA extraction (2). For estimation of library preparation efficiency and to address background contamination, nucleic acid spike-ins can be performed before molecular processing (3). PMA, propidium monoazide; PP, planetary protection; rRNA, ribosomal RNA.

Based on prior results and current technologies, experts at the workshop identified a hybrid molecular approach, combining nucleic acid extraction, quantitative digital PCR, and metagenomic sequencing as a strategy to both meet rapid enumeration goals and provide taxonomic and functional gene sequence data needed for sophisticated risk assessment. Using rapid nucleic acid extraction followed by digital PCR with domain-level and taxon-specific digital PCR assays, spacecraft-relevant microorganisms can be enumerated on the scale of 6 h (Fig. 1).

A slower metagenomic sequencing approach can be used for broad detection of microbial taxa and genes of concern and provide data for risk modeling. Overall, the desired outcome is a protocol that will be adopted into a NASA PP handbook and will include SOPs for nucleic acid extraction, decision-making protocol for selection of digital PCR assays, viable organism enumeration, and nucleic acid sequencing relevant to spacecraft bioburden analysis.

However, there is a need to invest resources and fund commercial development of methods to increase the yield of biomolecules during extractions and reduce reagent contamination (Minich *et al.*, 2018). Workshop experts also identified a need for DNA library synthesis protocols for long-read sequencers from ultra-low bioburden samples to improve identification of microbial taxa and genes.

### 5.3. Computational and bioinformatics analysis

Although the study of metagenomes is more than 20 years old (Handelsman *et al.*, 1998), relatively little effort has been made to standardize or assess the accuracy of the numerous software packages that perform quality control or annotation of sequence data, especially with low-biomass samples such as those collected from cleanroom surfaces (*e.g.*, McIntyre *et al.*, 2017; Vollmers *et al.*, 2017; Ye *et al.*, 2019). When comparing results from metagenomic analyses performed by different bioinformatics groups on the same dataset sampled from a cleanroom, Wood *et al.* (2021) demonstrated that there was poor agreement of detected species, and relative abundance of overlapping species.

Although each of the bioinformatics groups in the study detected species that have the potential for forward contamination, the disagreement in species-level annotation hampers the ability of metagenomic sequencing to provide actionable information to NASA. For example, falsely detected species might require extra cleaning of spacecraft, which could negatively affect the schedule of a spacecraft assembly and delay launch.

Conversely, problematic species that are present but not properly annotated could enable forward contamination. Most concerning was that none of the tested bioinformatics pipelines could annotate more than 15% of the sequence data, due to reference databases lacking the breadth of microbial diversity present in cleanroom environments or being of PP-relevance.

To address these concerns, significant effort is needed to test for accuracy (*i.e.*, specificity and sensitivity) and to standardize metagenomics pipelines, potentially using artificially generated metagenomes containing PP relevant taxa. To further increase the accuracy of these pipelines, public reference databases must be fortified with genomes of microbial species found in cleanroom environments, cleanroom-adjacent facilities without strict cleaning regimes (to capture hitchhikers), and from environments that resemble those on extraterrestrial planetary bodies (*e.g.*, radionucleotide dumping sites, nuclear accidents, and hot and cold arid biomes, etc.), before they are used for PP analysis.

In addition, database curation to remove poorly sequenced entries and/or to increase the stringency and accuracy of metagenomically assembly genomes (MAGs) is paramount to ensure accurate results. Efforts must be taken to maintain and expand functional gene databases relevant to classifying “contaminant risk” microorganisms. This could include maintaining a database of genes extracted from microorganisms that survive in high-risk environments and the construction of ortholog gene catalogs for target threat genes.

For example, there has been extensive research on physiological traits from Earth environments that are relevant for specific mission types, such as cold tolerance, endospore formation, UV tolerance, heat tolerance, radiation tolerance, etc., but PP-relevant data from these studies are curated in institutional databases and online repositories and have not been linked to PP compliance verification.

Current bioinformatic pipelines exist for spacecraft sequence data processing but require skilled computational biologists to operate. Streamlined reporting does exist for some metagenomic applications and could be adapted to analyze and report PP data. In addition, all workflows will need to be open access and transparent to allow for evaluation and reproducibility across a broad international scientific community.

In addition, sequence technologies that produce longer read lengths, such as Oxford Nanopore, should be qualified for use in low-biomass cleanroom environments since they are more likely to encode phylogenetically resolvable DNA (*e.g.*, Pearman *et al.*, 2020), and they may be less likely to represent background reagent contamination (*e.g.*, Olm *et al.*, 2017).

**RECOMMENDATION:** The computational resources need to include easy-to-use and revisable bioinformatics pipelines and output interfaces that meet the following requirements: (1) one-click bioinformatics analysis software solution for rapid assessment of PP-risk from metagenomic sequence data; (2) broad spectrum of PP-relevant microorganisms identified with defined confidence level analytics; (3) broad spectrum of space exploration-relevant functional gene content identified with defined confidence level analytics; (4) ability to generate metagenome-assembled genomes (MAGs) and enhance reference databases; (5) resolution framework for novel sequences; and (6) integrated noise reductions or background subtraction to compensate for contaminating nucleic acids from reagents.

Deliverables for achieving this goal include a comprehensive molecular analysis pipeline to enable real-time implementation on NASA missions. This includes an end-to-end systems level test with an easily executable pipeline and a graphical reporting interface. This system will depend on a curated database with known spacecraft assembly microorganisms and will be iteratively updated with *de novo* generated nucleic acid sequence data similar to the *Catalog of Earth's Microbiomes* (Nayfach *et al.*, 2021).

For each mission, the reference database will have to be fixed to ensure standardization of protocols. The bioinformatics must enable the generation of metagenome-assembled genomes (MAGs) and resolve novel sequences. The computational analysis also requires background noise assessment, including full characterization of reagent negative controls and molecular analyses of reagent lot numbers to characterize the kitomes used in the analysis. A reagent contamination database may be needed to fully address kitome issues.

### 5.4. PP-relevant microorganism assessment

For quantitative microbial risk assessment modeling, profiling of genetic traits of microbial communities is more valuable than phylogenetic identification. For example, identifying the presence of radiation resistance traits is critical, either through identification of specific organisms and inferring survival potential or through identification of resistance-relevant genes that can exist in multiple organisms. Similarly, genes encoding physiological capabilities of interest (*e.g.*, sporulation, anaerobiosis, desiccation resistance, extreme temperature resistance, and tolerance of other anticipated hostile spaceflight-relevant conditions) should be identified and included as PP-relevant genes in a risk analysis model.

Danko *et al.* (2021) noted, however, that although certain gene functions are critical for survival during spaceflight, these genes are not necessarily restricted to organisms that are PP-relevant (*e.g.*, DNA repair genes), and that bioinformatic- or culture-dependent analyses (where possible) should be performed to assess any given microorganism's resistance to radiation, desiccation, or other forces present in spaceflight.

Thus, although identification of PP-relevant genetic traits is critical, the detection of microbial taxa that are frequently identified in space programs should also be included in the relevant genetic feature list.

The abundance of genetic traits of concern is another important parameter for risk quantification, since risk scales with concentration. Despite the low biomass in these sample types and the elevated potential for background contamination, the effect of trace contamination in molecular analyses may be mitigated through highly targeted quantitative digital PCR assays and by demonstrating the potentially low impact on risk estimation of known reagent contaminants that do not have traits of PP relevance.

**RECOMMENDATION:** The goal of the PP relevant microbial assessment is to identify, enumerate, and sequence microorganisms that could survive and proliferate under conditions encountered in planetary exploration, and to contribute complete and draft genomes of taxa with PP-relevant physiological capabilities to reference databases. Relevant traits include desiccation survival (assembly, test, launch, and cruise), radiation/anaerobic tolerance (Mars and Jovian moons), psychrophily (Icy Worlds and spacecraft-induced habitable zones), and thermotolerance (de-orbit anomaly scenarios).

Workshop panelists noted that the International Space Station (ISS) microbial monitoring workflows, including molecular processing and cultivation already developed, can benefit PP policy and practices. Deliverables for the ISS monitoring goal include spacecraft PP risk models that can target relevant microorganisms (not all viable microorganisms) and an Earth-based extreme environment database of microorganisms, genomes, and functional genes. Similar databases highlighting microbial taxa relevant to planetary missions are also needed.

## 6. Recommendations and Outcomes from the Workshop

Panel experts agreed that there is a clear opportunity to incorporate molecular assays into the NASA PP efforts and identified a viable workflow to provide an unbiased assessment of the composition and gene content of microbial communities and quantitative analysis of bioburden. For missions in which forward and backward contamination are of concern, metagenome sequencing can provide all-inclusive phylogenetic and functional characteristics of the microorganisms present and supplement current cultivation-based approaches.

When rapid and quantitative measurements are needed, quantitative digital PCR was strongly recommended. The two methods can work in tandem, with metagenomics informing the design and application of digital PCR assays (Fig. 1). NASA's Office of Planetary Protection should establish a set of requirements that adhere to and expand upon established policies for microbial detection, microbial load assessment, and risk. The current suite of assays (NSA, ATP, LAL) has established quantitative requirements in specific applications that define detection levels/limits and PP risk.

The same approach is needed to introduce new molecular assays. Of particular importance is establishing efficacy and limits of detections for each step, evaluating and mitigating background nucleic acids reagent contamination. Collaboration with industry partners through NASA RFPs must be initiated to produce ultra-clean DNA-free reagents for DNA extraction, digital PCR, and library synthesis (*e.g.*, sample collection devices, water, nucleic acid extraction reagents, oligonucleotides, PCR master mixes, pre-amplification reagents, library synthesis reagents, etc.). To control for and mitigate signal noise, the development of required workflows should incorporate PP-relevant whole cell microbial reference controls at appropriate cell numbers. DNA controls at low input levels are also needed.

### 6.1. Specific recommendations of the Multi-Mission Metagenomics Technology Development Workshop Include:

#### 6.1.1. Sample collection

1. Nucleic acids free sampling devices and materials that are compatible with spacecraft components (*e.g.*, vacuum-based or tape-lifting methods, etc.) need to be identified.
2. Sampling strategies to recover PP-relevant microorganisms from small (25 cm^2^) and large (1 m^2^) surface areas are needed.

#### 6.1.2. Sample processing/sequencing

1. Nucleic acids-free reagents and consumables, including extraction reagents, PCR reagents, whole genome amplification reagents, oligonucleotides, and next-generation library synthesis reagents, are needed.
  i. Ensure workflows can be semi-automated using commercial nucleic acid extraction devices and library synthesis liquid handling robotics.
  ii. Identify alternative approaches to addressing reagent contamination, including but not limited to fragmentation of contaminant DNA through acoustic shearing, nucleic acid inactivation through cross-linking, deamination, etc.
  iii. Establish negative control sampling and processing strategy for minimizing and identifying reagent contamination.
2. Evaluate microorganism viability testing at low bioburden levels by comparing direct RNA extraction and sequencing with PMA pre-treatment or other nucleic acid inactivation protocols.
3. Develop ultra-low DNA and whole cell intact microbial reference standards for nucleic acid extraction and library synthesis efficiency assessment.
  i. Establish microbial and metagenomic standards that are recognized by federal agencies such as NIST and the International Metagenomics and Multiomics Standards Alliance (IMMSA).
  ii. Develop whole cell microbial reference standards that mimic true cells.
4. Evaluate high-efficiency, low nucleic acid input metagenomics library synthesis protocols for ultralow biomass samples (pg and sub-pg levels) and implement optimized workflow (*e.g.*, Minich *et al.*, 2018; Eisenhofer *et al.*, 2019).
  i. Develop library synthesis protocols for ultra-low biomass for long-read sequencing.

#### 6.1.3. Bioinformatic analysis

1. Establish a standard data submission pipeline to Genelab and the NCBI's Sequence Read Archive (SRA) with robust metadata, including non-nucleic acids-based bioburden data.
2. Evaluate open-source bioinformatics pipelines yielding robust taxonomic and functional gene assessment.
3. Develop one-stop, one-click bioinformatics analysis software package for risk assessment of metagenomic sequence data.
4. Validate bioinformatic pipeline using multi-institutional testing.
5. Develop a nucleic acids contamination reference database for all molecular biology reagents.

#### 6.1.4. Quantitative risk assessment of PP relevant microorganisms

1. Generate high-quality metagenome-assembled genomes (MAGs) from PP-relevant environmental samples.
2. Generate microbial genomes from isolated extremophilic microorganisms.
3. Establish reference databases of PP-relevant microorganisms and functional genes.
4. Establish a digital PCR assay library for PP-relevant microbial taxa and functional genes.
5. Develop a hybrid risk assessment model using metagenomics and quantitative digital PCR data.

It is important to consider timing when implementing new technologies into the NASA systems since there are many technical groups working in concert to accomplish critical tasks. The validation of new assays and workflows can take 4–5 years and will require a structured effort and adequate resources. To accomplish this, it will be necessary to act on the recommendations of the Committee on the Review of Planetary Protection Policy Development Processes of the SSB.

NASA should adequately fund both the Office of Planetary Protection and the research necessary to determine appropriate requirements for planetary bodies and to enable state-of-the-art PP techniques for monitoring and verifying compliance with these requirements. This should include periodic workshops to continuously evaluate technology development and implementation of new methods for bioburden assessment.

A significant amount of research has already been conducted by NASA in molecular and metagenomics analysis (Venkateswaran *et al.*, 2012; Danko *et al.*, 2021; Wood *et al.*, 2021) and building on those existing initiatives could validate this novel PP molecular approach by 2026. The development of metagenomic technologies from the current research-based environmental applications to a mainstream spacecraft application would be a step forward in modernizing NASA's PP program. This would benefit all future missions to Mars and the Icy Worlds by increasing the detection of microbial taxa with forward contamination potential and by providing comprehensive data reliability leading to reduced false positives in sample return, and ultimately, increased chance of scientific mission success.

In collaboration with the various groups and programs already leveraging sequencing or PCR-based approaches, NASA's Office of Planetary Protection needs to establish a set of requirements that adhere to and expand upon established policies for microbial detection, microbial load assessment, and risk calculation, and incorporate them in NASA PP Policy documents.

The current suite of assays (NSA, ATP, LAL) has established set requirements that define detection levels/limits and PP risk, and the same approach is needed to introduce a new molecular workflow and should not be delayed. The committee also recommends online maintenance of PP SOPs (*i.e.*, wiki format) that allows for global access and transparency, version tracking, topic queries, and focused community involvement.

## Abbreviations Used

ATP: adenosine triphosphate
COSPAR: Committee on Space Research
eDNA: environmental DNA
ISS: International Space Station
LAL: limulus amoebocyte lysate
MAG: metagenome-assembled genome
NSA: NASA Standard Assay
PP: planetary protection
PMA: propidium monoazide
rRNA: ribosomal RNA
SOP: standard operating procedure
SSB: Space Studies Board
VLP: virus like particles

## References

[B1] Ahsanuddin S, Afshinnekoo E, Gandara J, *et al.* (2017) Assessment of REPLI-g Multiple Displacement Whole Genome Amplification (WGA) techniques for metagenomic applications. J Biomol Tech 28:46–55. 28344519 10.7171/jbt.17-2801-008 PMC5363268

[B2] Angly FE, Dennis PG, Skarshewski A, *et al.* (2014) CopyRighter: a rapid tool for improving the accuracy of microbial community profiles through lineage-specific gene copy number correction. Microbiome 2:11. 24708850 10.1186/2049-2618-2-11 PMC4021573

[B3] Bargoma E, La Duc MT, Kwan K, *et al.* (2013) Differential recovery of phylogenetically disparate microbes from spacecraft-qualified metal surfaces. Astrobiology 13:189–202. 23421553 10.1089/ast.2012.0917

[B4] Bashir M, Ahmed M, Weinmaier T, *et al.* (2016) Functional metagenomics of Spacecraft Assembly Cleanrooms: presence of virulence factors associated with human pathogens. Front Microbiol 7:1321. 27667984 10.3389/fmicb.2016.01321 PMC5017214

[B5] Benardini JN and Venkateswaran K (2016) Application of the ATP assay to rapidly assess cleanliness of spacecraft surfaces: a path to set a standard for future missions. AMB Express 6:113. 27844457 10.1186/s13568-016-0286-9 PMC5108744

[B6] Buee M, Reich M, Murat C, *et al.* (2009) 454 Pyrosequencing analyses of forest soils reveal an unexpectedly high fungal diversity. New Phytol 184:449–456. 19703112 10.1111/j.1469-8137.2009.03003.x

[B7] Cooper M, La Duc MT, Probst A, *et al.* (2011) Comparison of innovative molecular approaches and standard spore assays for assessment of surface cleanliness. Appl Environ Microbiol 77:5438–5444. 21652744 10.1128/AEM.00192-11 PMC3147454

[B8] COSPAR (2011) COSPAR Planetary Protection Policy. World Space Council, Houston, TX, USA.

[B9] Danko DC, Sierra MA, Benardini JN, *et al.* (2021) A comprehensive metagenomics framework to characterize organisms relevant for planetary protection. Microbiome 9:82. 33795001 10.1186/s40168-021-01020-1 PMC8016160

[B10] de Goffau MC, Lager S, Salter SJ, *et al.* (2018) Recognizing the reagent microbiome. Nat Microbiol 3:851–853. 30046175 10.1038/s41564-018-0202-y

[B11] Eisenhofer R, Minich JJ, Marotz C, *et al.* (2019) Contamination in low microbial biomass microbiome studies: issues and recommendations. Trends Microbiol 27:105–117. 30497919 10.1016/j.tim.2018.11.003

[B12] Emerson JB, Adams RI, Roman CMB, *et al.* (2017) Schrodinger's microbes: tools for distinguishing the living from the dead in microbial ecosystems. Microbiome 5:86. 28810907 10.1186/s40168-017-0285-3 PMC5558654

[B13] Emiola A and Oh J (2018) High throughput in situ metagenomic measurement of bacterial replication at ultra-low sequencing coverage. Nat Commun 9:4956. 30470746 10.1038/s41467-018-07240-8 PMC6251912

[B14] Ghosh S, Osman S, Vaishampayan P, *et al.* (2010) Recurrent isolation of extremotolerant bacteria from the clean room where Phoenix spacecraft components were assembled. Astrobiology 10:325–335. 20446872 10.1089/ast.2009.0396

[B15] Green SJ, Venkatramanan R, and Naqib A. (2015) Deconstructing the polymerase chain reaction: understanding and correcting bias associated with primer degeneracies and primer-template mismatches. PLoS One 10:e0128122. 25996930 10.1371/journal.pone.0128122 PMC4440812

[B16] Handelsman J, Rondon MR, Brady SF, *et al.* (1998) Molecular biological access to the chemistry of unknown soil microbes: a new frontier for natural products. Chem Biol 5:R245–R249. 9818143 10.1016/s1074-5521(98)90108-9

[B17] Hendrickson R, Urbaniak C, Minich JJ, *et al.* (2021) Clean room microbiome complexity impacts planetary protection bioburden. Microbiome 9:238. 34861887 10.1186/s40168-021-01159-x PMC8643001

[B18] Hug LA, Baker BJ, Anantharaman K, *et al.* (2016) A new view of the tree of life. Nat Microbiol 1:16048. 27572647 10.1038/nmicrobiol.2016.48

[B19] Huse SM, Huber JA, Morrison HG, *et al.* (2007) Accuracy and quality of massively parallel DNA pyrosequencing. Genome Biol 8:R143. 17659080 10.1186/gb-2007-8-7-r143 PMC2323236

[B20] Kembel SW, Wu M, Eisen JA, *et al.* (2012) Incorporating 16S gene copy number information improves estimates of microbial diversity and abundance. PLoS Comput Biol 8:e1002743. 23133348 10.1371/journal.pcbi.1002743 PMC3486904

[B21] Kern, R., Kazarians, G., Beaudet, R.A., *et al.* (2005) Rapid Enzyme-Based Microbial Burden Assays: the Case for Certifying Total Adenosine Triphosphate Assay for Use on Spacecraft Hardware. JPL D-30970. Jet Propulsion Laboratory, California Institute of Technology, Pasadena, CA.

[B22] Klindworth A, Pruesse E, Schweer T, *et al.* (2013) Evaluation of general 16S ribosomal RNA gene PCR primers for classical and next-generation sequencing-based diversity studies. Nucleic Acids Res 41:e1. 22933715 10.1093/nar/gks808 PMC3592464

[B23] Kwan K, Cooper M, La Duc MT, *et al.* (2011) Evaluation of procedures for the collection, processing, and analysis of biomolecules from low-biomass surfaces. Appl Environ Microbiol 77:2943–2953. 21398492 10.1128/AEM.02978-10 PMC3126404

[B24] La Duc MT, Dekas A, Osman S, *et al.* (2007) Isolation and characterization of bacteria capable of tolerating the extreme conditions of clean room environments. Appl Environ Microbiol 73:2600–2611. 17308177 10.1128/AEM.03007-06 PMC1855582

[B25] La Duc MT, Osman S, and Venkateswaran K. (2009) Comparative analysis of methods for the purification of DNA from low-biomass samples based on total yield and conserved microbial diversity. J Rapid Methods Autom Microbiol 17:350–368; 1060–3999.

[B26] La Duc MT, Vaishampayan P, Nilsson HR, *et al.* (2012) Pyrosequencing-derived bacterial, archaeal, and fungal diversity of spacecraft hardware destined for Mars. Appl Environ Microbiol 78:5912–5922. 22729532 10.1128/AEM.01435-12 PMC3406123

[B27] La Duc MT, Venkateswaran K, and Conley CA. (2014) A genetic inventory of spacecraft and associated surfaces. Astrobiology 14:15–23. 24432775 10.1089/ast.2013.0966

[B28] Li R, Tun HM, Jahan M, *et al.* (2017) Comparison of DNA-, PMA-, and RNA-based 16S rRNA Illumina sequencing for detection of live bacteria in water. Sci Rep 7:5752. 28720878 10.1038/s41598-017-02516-3 PMC5515937

[B29] Loy A, Lehner A, Lee N, *et al.* (2002) Oligonucleotide microarray for 16S rRNA gene-based detection of all recognized lineages of sulfate-reducing prokaryotes in the environment. Appl Environ Microbiol 68:5064–5081. 12324358 10.1128/AEM.68.10.5064-5081.2002 PMC126405

[B30] Marotz CA, Sanders JG, Zuniga C, *et al.* (2018) Improving saliva shotgun metagenomics by chemical host DNA depletion. Microbiome 6:42. 29482639 10.1186/s40168-018-0426-3 PMC5827986

[B31] McIntyre AB, Ounit R, Afshinnekoo E, *et al.* (2017) Comprehensive benchmarking and ensemble approaches for metagenomic classifiers. Genome Biol 18:1–19. 28934964 10.1186/s13059-017-1299-7 PMC5609029

[B32] Minich JJ, Zhu Q, Janssen S, *et al.* (2018) KatharoSeq enables high-throughput microbiome analysis from low-biomass samples. mSystems 3 (3): e00218-17. 29577086 10.1128/mSystems.00218-17 PMC5864415

[B33] Morris HC, Monaco LA, Steele A, *et al.* (2010) Setting a standard: the limulus amebocyte lysate assay and the assessment of microbial contamination on spacecraft surfaces. Astrobiology 10:845–852. 21087163 10.1089/ast.2009.0446

[B34] Naqib A, Poggi S, and Green SJ. (2019) Deconstructing the Polymerase Chain Reaction II: an improved workflow and effects on artifact formation and primer degeneracy. Peer J 7:e7121. 31231597 10.7717/peerj.7121 PMC6573857

[B35] Nardello LCL, Vilela BG, Fernandes FS, *et al.* (2020) Analysis of active bacteria persisting after chemomechanical procedures: an RNA- and DNA-based molecular study. J Endod 46:1570–1576. 32805336 10.1016/j.joen.2020.08.004

[B36] NASA (1967) NHB5340.1 Standard Procedures for Microbial Examinations of Spacecraft Hardware. Washington, DC.

[B37] NASA (2011) Planetary Protection Provisions for Robotic Extraterrestrial Missions. NPR 8020.12D, April 2011. National Aeronautics and Space Administration, Washington, DC.

[B38] NASA (2019) NASA Policy Instruction-8020.7G: NASA Policy on Planetary Protection Requirements for Human Extraterrestrial Missions. NASA, Washington, DC.

[B39] National Academies of Sciences, Engineering, and Medicine (2021) Report Series: Committee on Planetary Protection: Evaluation of Bioburden Requirements for Mars Missions. The National Academies Press, Washington, DC. 35015394

[B40] Nayfach S, Roux S, Seshadri R, *et al.* (2021) A genomic catalog of Earth's microbiomes. Nat Biotechnol 39:499–509. 33169036 10.1038/s41587-020-0718-6 PMC8041624

[B41] Nocker A, Cheung CY, and Camper AK. (2006) Comparison of propidium monoazide with ethidium monoazide for differentiation of live vs. dead bacteria by selective removal of DNA from dead cells. J Microbiol Methods 67:310–320. 16753236 10.1016/j.mimet.2006.04.015

[B42] NRC (1992) Biological Contamination of Mars: Issues and Recommendations (ed.) NASA. NRC: New York.

[B43] NRC (2006) Preventing the Forward Contamination of Mars. Committee on Preventing the Forward Contamination of Mars. National Research Council. National Academies Press, Washington, DC.

[B44] Olm MR, Butterfield CN, Copeland A, *et al.* (2017) The source and evolutionary history of a microbial contaminant identified through soil metagenomic analysis. mBio 8:e01969-16. 28223457 10.1128/mBio.01969-16 PMC5358914

[B45] Pearman WS, Freed NE, and Silander OK. (2020) Testing the advantages and disadvantages of short- and long- read eukaryotic metagenomics using simulated reads. BMC Bioinformatics 21:220. 32471343 10.1186/s12859-020-3528-4 PMC7257156

[B46] Poretsky R, Rodriguez RL, Luo C, *et al.* (2014) Strengths and limitations of 16S rRNA gene amplicon sequencing in revealing temporal microbial community dynamics. PLoS One 9:e93827. 24714158 10.1371/journal.pone.0093827 PMC3979728

[B47] Rinke C, Low S, Woodcroft BJ, *et al.* (2016) Validation of picogram- and femtogram-input DNA libraries for microscale metagenomics. PeerJ 4:e2486. 27688978 10.7717/peerj.2486 PMC5036114

[B48] Salter SJ, Cox MJ, Turek EM, *et al.* (2014) Reagent and laboratory contamination can critically impact sequence-based microbiome analyses. BMC Biol 12:87. 25387460 10.1186/s12915-014-0087-z PMC4228153

[B49] Schnetzinger F, Pan Y, and Nocker A (2013) Use of propidium monoazide and increased amplicon length reduce false-positive signals in quantitative PCR for bioburden analysis. Appl Microbiol Biotechnol 97:2153–2162. 23354451 10.1007/s00253-013-4711-6

[B50] Silander K and Saarela J (2008) Whole genome amplification with Phi29 DNA polymerase to enable genetic or genomic analysis of samples of low DNA yield. Methods Mol Biol 439:1–18. 18370092 10.1007/978-1-59745-188-8_1

[B51] Singh NK, Wood JM, Karouia F, *et al.* (2018) Succession and persistence of microbial communities and antimicrobial resistance genes associated with International Space Station environmental surfaces. Microbiome 6:204. 30424821 10.1186/s40168-018-0585-2 PMC6234677

[B52] Tan C, Fan D, Wang N, *et al.* (2021) Applications of digital PCR in COVID-19 pandemic. View (Beijing): 20200082. 34766158 10.1002/VIW.20200082 PMC7883284

[B53] UNOOSA (2017) *Report of the Committee on the Peaceful Use of Outer Space*, 60th A/72/20.

[B54] Vaishampayan P, Probst AJ, La Duc MT, *et al.* (2013) New perspectives on viable microbial communities in low-biomass cleanroom environments. ISME J 7:312–324. 23051695 10.1038/ismej.2012.114 PMC3554398

[B55] Venkateswaran K, La Duc MT, and Vaishampayan, P. (2012) Genetic Inventory Task: Final Report, JPL Publication 12-12. Jet Propulsion Laboratory, California Institute of Technology: Pasadena, CA.

[B56] Vollmers J, Wiegand S, and Kaster AK. (2017) Comparing and Evaluating Metagenome Assembly Tools from a microbiologist's perspective—not only size matters! PLoS One 12:e0169662. 10.1371/journal.pone.0169662 PMC5242441 28099457

[B57] Wang Y, Yan Y, Thompson KN, *et al.* (2021) Whole microbial community viability is not quantitatively reflected by propidium monoazide sequencing approach. Microbiome 9:17. 33478576 10.1186/s40168-020-00961-3 PMC7819323

[B58] Wood JM, Singh NK, Guan L, *et al.* (2021) Performance of multiple metagenomics pipelines in understanding microbial diversity of a low-biomass spacecraft assembly facility. Front Microbiol 12:685254. 34650522 10.3389/fmicb.2021.685254 PMC8508200

[B59] Ye SH, Siddle KJ, Park DJ, *et al.* (2019) Benchmarking metagenomics tools for taxonomic classification. Cell 178:779–794. 31398336 10.1016/j.cell.2019.07.010 PMC6716367

